# Supplementary material for: Cross-shore parallel tidal channel systems formed by alongshore currents
Source: Nat Commun. 2024 Jun 3;15:4732. doi: 10.1038/s41467-024-49176-2 (PMC11148050; doi:10.1038/s41467-024-49176-2)
Supplement: Supplementary file 1 — Supplementary Information [file 41467_2024_49176_MOESM1_ESM.pdf]

Supplementary Information for
Cross-shore parallel tidal channel systems formed by alongshore currents

Zeng Zhou[1,2], Yizhang Wei[2], Liang Geng[1,3*], Ying Zhang[2,4], Yuxian Gu[2], Alvise Finotello[5], Andrea D'Alpaos[5], Zheng Gong[1], Fan Xu[3*], Changkuan Zhang[2], Giovanni Coco[6]

---

[1] The National Key Laboratory of Water Disaster Prevention, Hohai University, Nanjing 210024, China

[2] Jiangsu Key Laboratory of Coastal Ocean Resources Development and Environment Security, Hohai University, Nanjing 210024, China

[3] State Key Laboratory of Estuarine and Coastal Research, East China Normal University, Shanghai 200062, China

[4] Bureau of Water Resources of Luhe District, Nanjing 211500, China

[5] Department of Geosciences, University of Padova, Padova, Italy.

[6] Faculty of Science, University of Auckland, Private Bag 92019, Auckland, New Zealand.

* Corresponding authors: gengliang1991@hhu.edu.cn and fxu@sklec.ecnu.edu.cn.

Supplementary Methods

Section 1: Determination of tidal channel angles.

The locations, coastal conditions, and average channel angles of 21 typical parallel tidal channel systems worldwide are recorded in Table S1.1. The definition and size of the angles of each channel are shown in Figure S1.1.

Table S1.1. Site characteristics of the selected locations for parallel channel analysis

Site	Latitude, longitude (decimal degrees)	Averaged intersecting angle α (°)	Averaged overall angle β (°)	Tidal currents	Type
Chongming Island, China	31°42'N, 121°38'E	91.6	85	Alongshore[1,2]	Estuary
Warbah Island, Kuwait	30°1'N, 48°4'E	87	89.1	Alongshore[3,4]	Estuary
Tillingham Tidal Flats, UK	51°40'N, 0°56'E	78.25	84.1	Alongshore	Open coast
Hooge Plates, Netherlands	51°23'N, 3°39'E	83.5	92.7	Alongshore[5]	Estuary
Bath Tidal Flats Western Scheldt, Netherlands	51°22'N, 4°14'E	83.4	83.2	Alongshore[6]	Estuary
Ems-Dollard, Netherlands	53°21'N, 6°54'E	97	106.3	Alongshore[7]	Lagoon
San Francisco Bay, USA	37°27'N, 122°1'W	98	93.5	Alongshore[8]	Lagoon
Meda River Delta, Australia	17°2'S, 123°54'E	79.1	82.3	Alongshore[9]	Lagoon

Jiangsu Coast, China	32°59'N, 120°55'E	104.8	105	Alongshore	Open coast
Orne Estuary, France	49°16'N, 0°14'W	92	92.8	Alongshore	Estuary
Inhassunge Tidal Flats, Mozambique	18°10'S, 36°50'E	71	65.8	Alongshore	Lagoon
Iranian Bay, Iran	30°11'N, 49°11'E	71.8	79.5	Alongshore[3,4]	Estuary
Shoalwater Bay, Australia	22°28'S, 150°33'E	41.3	78.5	Alongshore	Open coast
Bahmanshir River Mouth, Iran	30°1'N, 48°41'E	61.3	61.2	Alongshore[3,4]	Estuary
Taeryong River Delta, North Korea	39°35'N, 125°13'E	51	65.8	Alongshore	Estuary
Rio Cabelo da Valha, Brazil	1°43'S, 44°46'W	67.8	100.3	Alongshore	Estuary
Thengar Island, Bangladesh	22°32'N, 91°30'E	71.9	79.2	Alongshore	Estuary
Colorado River Delta, Mexico	31°47'N, 114°48'W	87.4	63.9	Alongshore	Estuary
Bahia Blanca, Argentina	38°48'S, 62°18'W	67.3	108.2	Alongshore	Lagoon
Rio Cacine Channel, Guinea	11°9'N, 15°3'W	68	72.4	Alongshore	Lagoon
Broome Tidal Flats, Australia	17°59'S, 122°21'E	102.7	74	Alongshore	Open coast

25

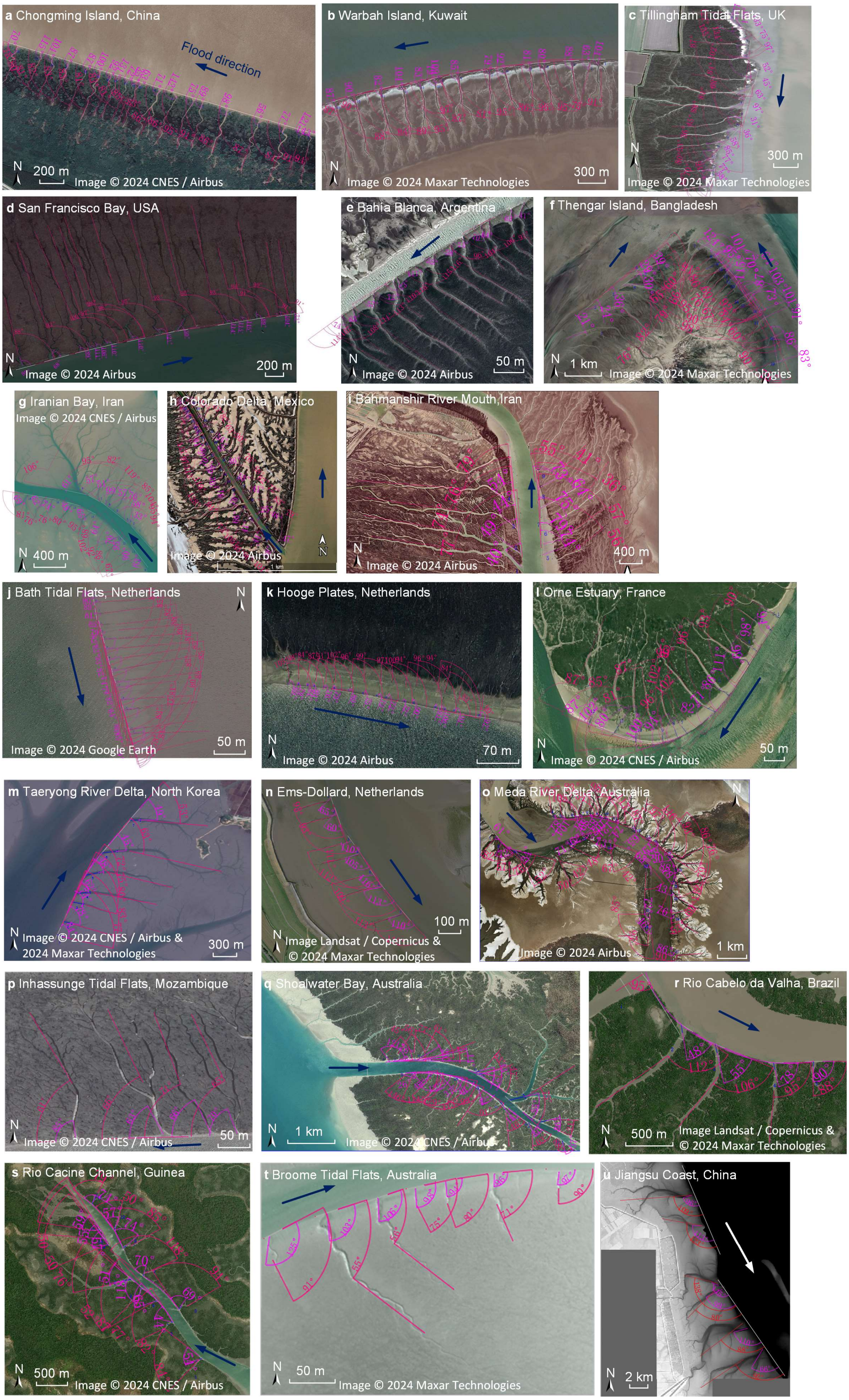
a Chongming Island, China
Flood direction
200 m
Image © 2024 CNES / Airbus
b Warbah Island, Kuwait
300 m
Image © 2024 Maxar Technologies
c Tillingham Tidal Flats, UK
300 m
Image © 2024 Maxar Technologies
d San Francisco Bay, USA
Image © 2024 Airbus
200 m
e Bahia Blanca, Argentina
50 m
Image © 2024 Airbus
f Thengar Island, Bangladesh
1 km
Image © 2024 Maxar Technologies
g Iranian Bay, Iran
Image © 2024 CNES / Airbus
400 m
h Colorado Delta, Mexico
Image © 2024 Airbus
i Bahmanshir River Mouth, Iran
Image © 2024 Airbus
400 m
j Bath Tidal Flats, Netherlands
Image © 2024 Google Earth
50 m
k Hooge Plates, Netherlands
Image © 2024 Airbus
70 m
l Orne Estuary, France
Image © 2024 CNES / Airbus
50 m
m Taeryong River Delta, North Korea
Image © 2024 CNES / Airbus &
2024 Maxar Technologies
300 m
n Ems-Dollard, Netherlands
100 m
Image Landsat / Copernicus &
© 2024 Maxar Technologies
o Meda River Delta, Australia
Image © 2024 Airbus
1 km
p Inhassunge Tidal Flats, Mozambique
Image © 2024 CNES / Airbus
50 m
q Shoalwater Bay, Australia
1 km
Image © 2024 CNES / Airbus
r Rio Cabelo da Valha, Brazil
500 m
Image Landsat / Copernicus &
© 2024 Maxar Technologies
s Rio Cacine Channel, Guinea
500 m
Image © 2024 CNES / Airbus
t Broome Tidal Flats, Australia
50 m
Image © 2024 Maxar Technologies
u Jiangsu Coast, China
2 km

Figure S1.1. Overview of 21 systems analysed for channel angles. a Chongming Island, China, Image © 2024 CNES / Airbus. **b** Warbah Island, Kuwait, Image © 2024 Maxar Technologies. **c** Tillingham Tidal Flats, UK, Image © 2024 Maxar Technologies. **d** San Francisco Bay, USA, Image © 2024 Airbus. **e** Bahía Blanca, Argentina, Image © 2024 Airbus. **f** Thengar Island, Bangladesh, Image © 2024 Maxar Technologies. **g** Iranian Bay, Iran, Image © 2024 CNES / Airbus. **h** Colorado Delta, Mexico, Image © 2024 Airbus. **i** Bahmanshir River Mouth, Iran, Image © 2024 Airbus. **j** Bath Tidal Flats, Netherlands, Image © Google Earth. **k** Hooge Plates, Netherlands, Image © 2024 Airbus. **l** Orne Estuary, France, Image © 2024 CNES / Airbus. **m** Taeryong River Delta, North Korea, Image © 2024 CNES / Airbus & 2024 Maxar Technologies. **n** Ems-Dollard, Netherlands, Image Landsat / Copernicus & © 2024 Maxar Technologies. **o** Meda River Delta, Australia, Image © 2024 Airbus. **p** Inhassunge Tidal Flats, Mozambique, Image © 2024 CNES / Airbus. **q** Shoalwater Bay, Australia, Image © 2024 CNES / Airbus. **r** Rio Cabelo da Valha, Brazil, Image Landsat / Copernicus & © 2024 Maxar Technologies. **s** Rio Cacine Channel, Guinea, Image © 2024 CNES / Airbus. **t** Broome Tidal Flats, Australia, Image © 2024 Maxar Technologies. **u** Jiangsu Coast, China, originates from LiDAR survey data (2006). Tidal channel angle is measured as the angle between the parallel branch and the shoreline at the low water level, consistent with the flood flow direction, as shown by the purple numbers in the figure.

As can be seen from Figure S1.1, the degree of curvature of tidal channel branches in different parallel tidal channel systems is also different. In the main text, we do not focus on the morphology of a single branch, but focus on the morphological characteristics of the parallel tidal channel system as a whole. Therefore, in this Supplementary Information, we briefly discuss the factors influencing the bend shape of a single tidal channel.

In the field of river studies, it has been observed that channels in areas with steep slopes are typically straight, whereas those in gently sloping regions exhibit more meandering patterns, as shown in Figure S1.2a. Lazarus and Constantine (2013) attempted to provide a general explanation, suggesting that a higher Froude number reduces the degree of meandering[10]. The Froude number represents the ratio of hydraulic slope to flow resistance. Lower Froude numbers imply increased friction, resulting in greater resistance to channel flow. Under these conditions, the system, striving to enhance drainage efficiency, opts for increased sinuosity to extend the channel length.

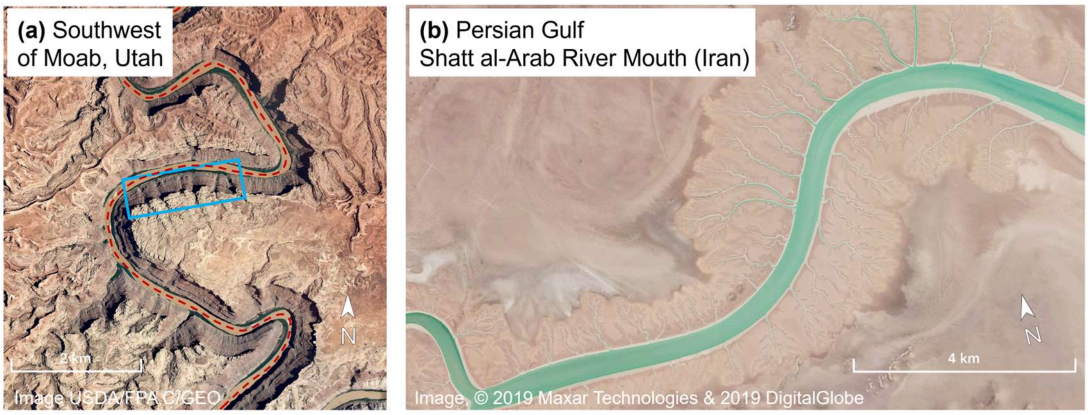

Figure S1.2. The comparison between terrain and coastal drainage systems. a The meandering river on the flat terrain (marked by the red dashed line) and the straight

gullies on the cliffs on both sides (marked in a blue box) show the control of the hydraulic slope over the channel shape in the terrestrial environment. Source: Image USDA/FPA C/GEO. **b** The parent channel and the parallel channel systems in Persian Gulf, Shatt al-Arab River Mouth, Iran. Source: Image, © 2019 Maxar Technologies & 2019 DigitalGlobe.

This explanation extends to the tidal environments over mudflats, as discussed in our previously published work[11]. As shown in Figure S1.2b, the regular pattern of parallel channels does not extend far into the interior of the mudflat. This is because in the region adjacent to the main channel, where the flow is strong, the flow dynamics is similar to mountainous areas with a higher Froude number. On the other hand, the interior of mudflat is more consistent with the friction-dominant hypothesis proposed by Rinaldo et. al. (1999), and therefore exhibits a more meander pattern[12].

To further visualize this phenomenon, we compared the morphology of the tidal channel systems in Venice Lagoon, Italy and in Mokpo, west coast of South Korea (Figure S1.3). The Venice lagoon, characterized by its small tidal range and significant vegetation, demonstrates a clear friction dominance, resulting in a meandering channel morphology. Conversely, in the Korean region, where the tidal range is substantial, especially along the large tidal channels, the friction component is relatively lower, leading to the pronounced development of straight, parallel channels.

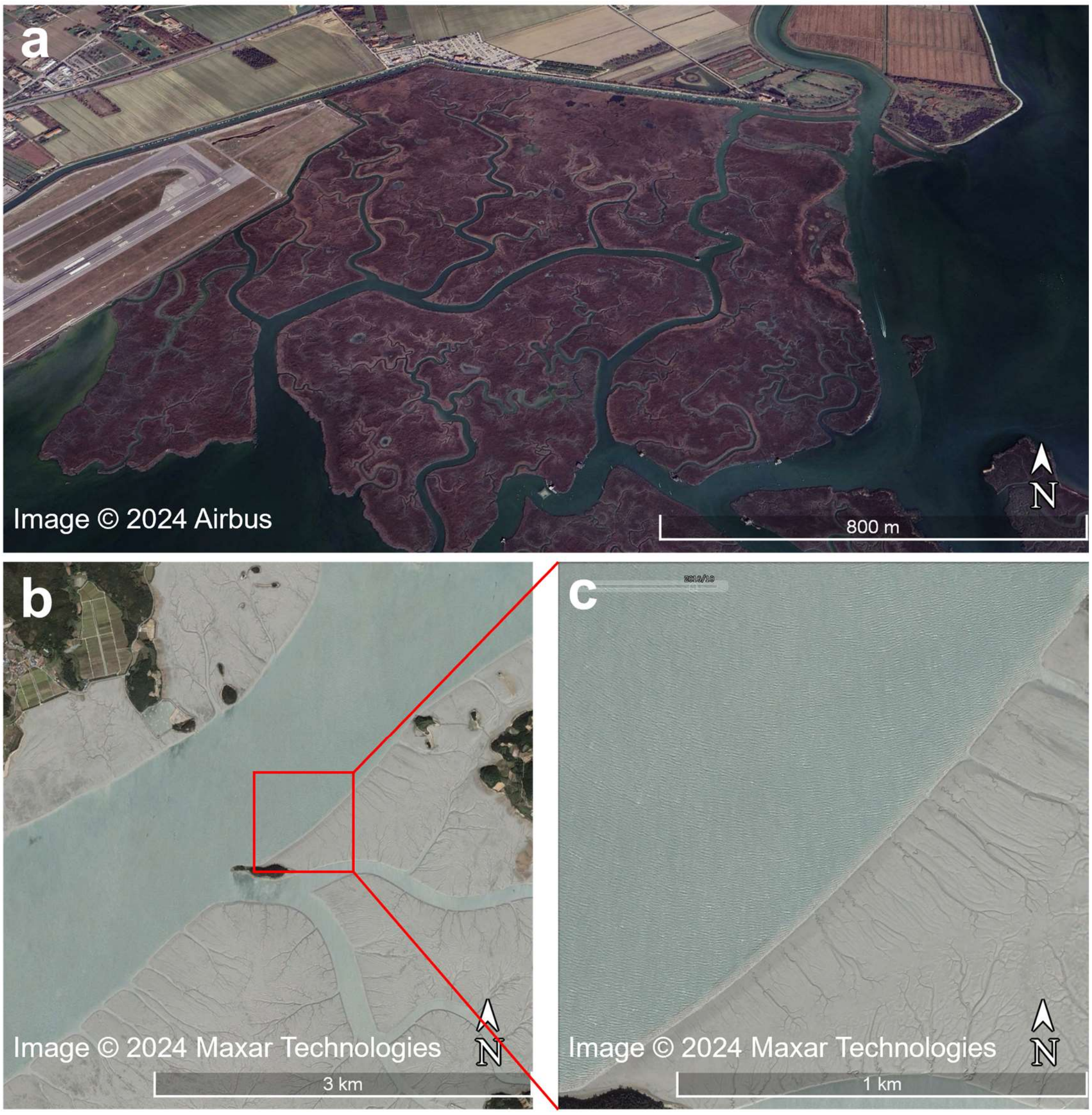

Figure S1.3. The comparison between two typical channel systems. a The meandering tidal channels observed in the Venice Lagoon, characterized by small tidal ranges and vegetation. Source: Image © 2024 Airbus. **b&c** Straight tidal channels observed on the west coast of Korea, characterized by strong tides and absence of vegetation. The difference between them highlights the controlling effects of friction on channel pattern. Source: Image © 2024 Maxar Technologies.

Section 2: Collection of environmental factors in the study areas

Table. S2.1. Background information on reference field sites in southeastern China (Chongming Island and Jiangsu Coast)

	Chongming Island	Jiangsu Coast
	Perpendicular PCs	Non-perpendicular PCs
**Hydrodynamics**		
Mean tidal range (cm)	307[13]	368[14]
**Sediment: value (Year)**		
Grain size d50 (μm)	104 (2012)[15]	81 (2003)[16]
Sand content (%)	57%-71% (2016)[15]	83%-98% (2003)[16]
Clay content (%)	28%-43% (2016)[15]	2%-17% (2003)[16]
**Slope (mm^{-1}): value (Year)**		
Channel area	0.0006 (2019)	0.0004 (2022)
Subtidal area	0.002 (2019)	0.0008 (2022)

Table. S2.2. Sedimentology information on reference field sites

**Site**	**D50 (μm)**	**Sand (%)**	**Silt (%)**	**Clay (%)**
Chongming Island, China[15]	104	57-71%		28-43%
Warbah Island, Kuwait[17,18]	7.50 phi	3%	54%	43%
Tillingham Tidal Flats, UK[19-21]	43	<10%	40-60%	25-45%
Hooge Plates, and Bath Tidal Flats Western Scheldt, Netherlands[22-24]	20~135	predominant	<3%	<3%
Ems-Dollard, Netherlands[25]	152	mean 87%	<13%	<13%
San Francisco Bay, USA[26,27]	100~500		predominant	
Meda River Delta, Australia[28]	///	///	mud predominant	
Jiangsu Coast, China[16]	81	83-98%		2%-17%
Orne Estuary, France[29]	3 phi	predominant		
Inhassunge Tidal Flats, Mozambique	///	///	///	///
Iranian Bay, Iran- ---no journals found	///	///	///	///
Shoalwater Bay, Australia[30]	///	>90%	<5%	<5%
Bahmanshir River Mouth, Iran	///	///	///	///
Taeryong River Delta, North Korea ---few journals	///	///	///	///
Rio Cabelo da Valha, Brazil- ---no journals found	///	///	///	///
Thengar Island, Bangladesh ---few journals	///	///	///	///
Colorado River Delta, Mexico				
Bahia Blanca, Argentina				
Rio Cacine Channel, Guinea---few journals	///	///	///	///
Broome Tidal Flats, Australia				

Section 3: Model description and scenarios of numerical experiments

Table. S3.1. Hydro-morphodynamic model equations

Hydro-morphodynamic processes		
Nr.	Governing equation	Short description
(1)	$\frac{\partial \eta}{\partial t} + \frac{\partial hu}{\partial x} + \frac{\partial hv}{\partial y} = 0$ $\frac{\partial u}{\partial t} + u\frac{\partial u}{\partial x} + v\frac{\partial u}{\partial y} = fv - g\frac{\partial \eta}{\partial x} + \nu\left(\frac{\partial^2 u}{\partial x^2} + \frac{\partial^2 u}{\partial y^2}\right) - g\frac{v\sqrt{u^2+v^2}}{C^2 h}$ $\frac{\partial v}{\partial t} + u\frac{\partial v}{\partial x} + v\frac{\partial v}{\partial y} = -fu - g\frac{\partial \eta}{\partial y} + \nu\left(\frac{\partial^2 v}{\partial x^2} + \frac{\partial^2 v}{\partial y^2}\right) - g\frac{v\sqrt{u^2+v^2}}{C^2 h}$	shallow water equations
(2)	$\frac{\partial (ch)}{\partial t} + \frac{\partial (uch)}{\partial x} = Q_{mud,e} - Q_{mud,d}$ $Q_{mud,e} = \begin{cases} M_e\left(\frac{\tau_{max}}{\tau_{cr,e}}\right) - 1, & if\ \tau_{max} > \tau_{cr,e} \\ 0, & if\ \tau_{max} \leq \tau_{cr,e} \end{cases}$ $Q_{mud,d} = \begin{cases} w_s c\left(1 - \frac{\tau_{max}}{\tau_{cr,d}}\right), & if\ \tau_{max} < \tau_{cr,d} \\ 0, & if\ \tau_{max} \geq \tau_{cr,d} \end{cases}$ $S_{sand} = A_s u\left[(u^2 + \frac{0.018}{C_{dc}} U_{rms}^2)^{1/2} - u_{cr}\right]^{2.4}$	Sediment transport formulations
(3)	$(1-\varepsilon)\frac{\partial z}{\partial t} + \frac{\partial S_x}{\partial x} + \frac{\partial S_y}{\partial y} = 0$	Bed level update

where u and v are the depth-averaged velocities in x and y directions (m/s), respectively; t is time (s); g is the gravitational acceleration (m/s^2); f is the Coriolis force coefficient (1/s); h is the water depth (m); η is the water level with respect to datum (m); ν is the eddy viscosity coefficient (m^2/s); C is the Chézy friction coefficient (m$^{1/2}$/s); τ_{max} is the maximum bed shear stress (Pa); $Q_{mud,e}$ and $Q_{mud,d}$ are respectively erosion and deposition fluxes described by the widely-adopted Partheniades–Krone formulations; M_e is the erosion parameter (kg/m^2/s); w_s is the settling velocity (m/s); c is the depth-averaged concentration (kg/m^3); $\tau_{cr,e}$ and $\tau_{cr,d}$ are the critical shear stress for erosion and deposition of mud fraction, respectively (Pa); S_{sand} is the total sediment transport of sand particles (m^2/s); As is the parameter related to sediment properties and water depth; C_{dc} is a non-dimensional drag coefficient due to current alone; U_{rms} is the root-mean-square wave orbital velocity that can be related to wave-orbital speed (m/s); u_{cr} is the threshold velocity for sediment mobilization and is estimated as a function of sediment grain size (m/s); ε is bed porosity; z is bed level; S_x and S_y are sediment transports in x and y directions, respectively.

Table. S3.2. Hydro-morphodynamic model parameters

Symbol	Parameter	Unit	Value	Ref.
t	hydrodynamic time step	min	0.5	
g	the gravitational constant	kg/s^2	9.81	31
ν	the horizontal eddy viscosity coefficient	m^2/s	1	31
M_e	the erosion parameter	kg/m^2/s	5×10^{-5}	32
w_s	the settling velocity	mm/s	0.5	32
$\tau_{cr,e}$	the critical shear stress for erosion	Pa	0.2	32
d50	median sediment diameter	μm	50	16

C_{dc}	non-dimensional drag coefficient due to current alone $C_{dc} = [0.4/(\ln\ h/z_r - 1)]^2$	-		[33]
z_r	the bed roughness length	m	0.006	[33]
ε	bed porosity	-	0.4	[34]
f_{mor}	morphological acceleration factor	-	25	

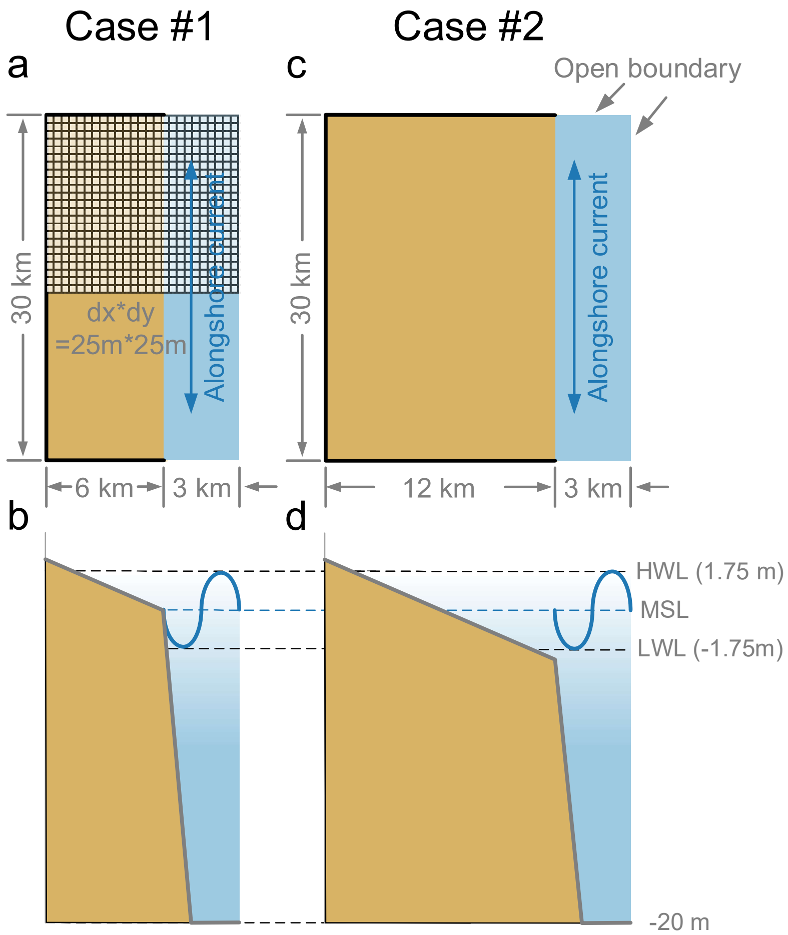

Figure. S3.1. Description of the model domain. a&b Simplified model domain of Chongming tidal flats, representing the intertidal zone with sharp transition. **c&d** model domain representing the schematic Jiangsu coast, with gentle slope changes in the intertidal zone.

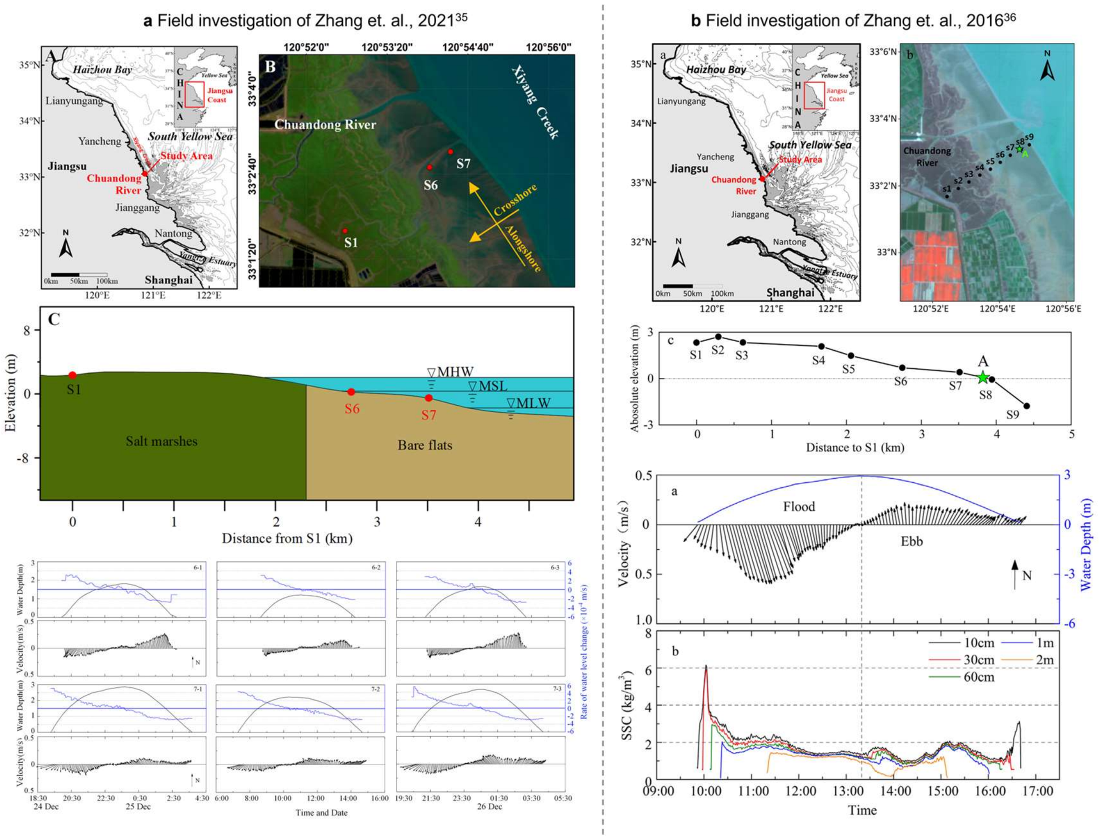

Figure S3.2. On-site observations of flow field in Jiangsu Coast[35,36]. a Field investigation and results of Zhang et. al., 2021[35], including the study area, bed profile, and the variations of water depth and velocity vector at S6 and S7. **b** Field investigation and results of Zhang et. al., 2016[36], including the study area, bed profile, and the variations of water depth, velocity vector, and suspended sediment concentration at station A. Reprinted from Continental Shelf Research, Vol 113, Zhang, Q. et al., 'Velocity and sediment surge: What do we see at times of very shallow water on intertidal mudflats?', Pages 10-20, Copyright (2016), with permission from Elsevier. All Rights Reserved.

As observed by Zhang et. al. (2016&2021)[35,36] in the Jiangsu Coast (shown in Fig. S3.2), the alongshore component of the velocity ellipse at the seaward station A, is much larger than the cross-shore one, indicating that the seaward area is dominated by the alongshore currents. Moving landward to stations S7 and S6, the cross-shore velocity component gradually increases, which is consistent with to the model results for the case of linearly-sloping bed.

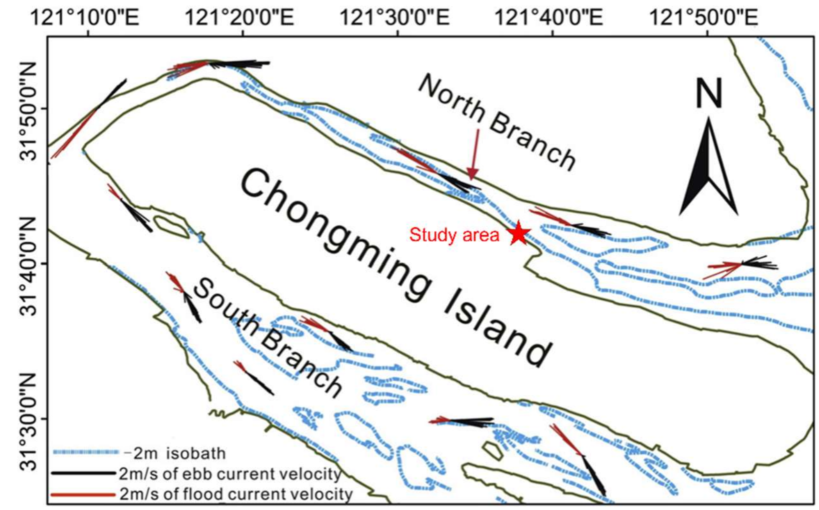

Figure S3.3. Spatial variations in flood/ebb current directions in the North Branch and South Branch (Synchronous survey date: 22 Sept. 2002)[37]. Reprinted from Marine Geology, Vol 379, Dai, Z., et al., 'Linking the infilling of the North Branch in the Changjiang (Yangtze) estuary to anthropogenic activities from 1958 to 2013', Pages 1-12, Copyright (2016), with permission from Elsevier. All Rights Reserved. The location of the parallel channel system in our study is marked by the red star.

Fig. S3.3 portrays the flow field measured in the field (Dai et. al., 2013)[37] in Chongming island, whose bed profile is characterized by a sharp transition in elevation at the bank of the north branch of Changjiang river. In the north branch of Changjiang river, the flow direction is mainly along the channel, and the transversal velocity is restricted by the channel bank, consistent with the channel velocity in the simulated scarped-shaped case (see Fig. 3a in the main text).

Supplementary Discussion

Section 4: Analysis of parallel channel characteristics

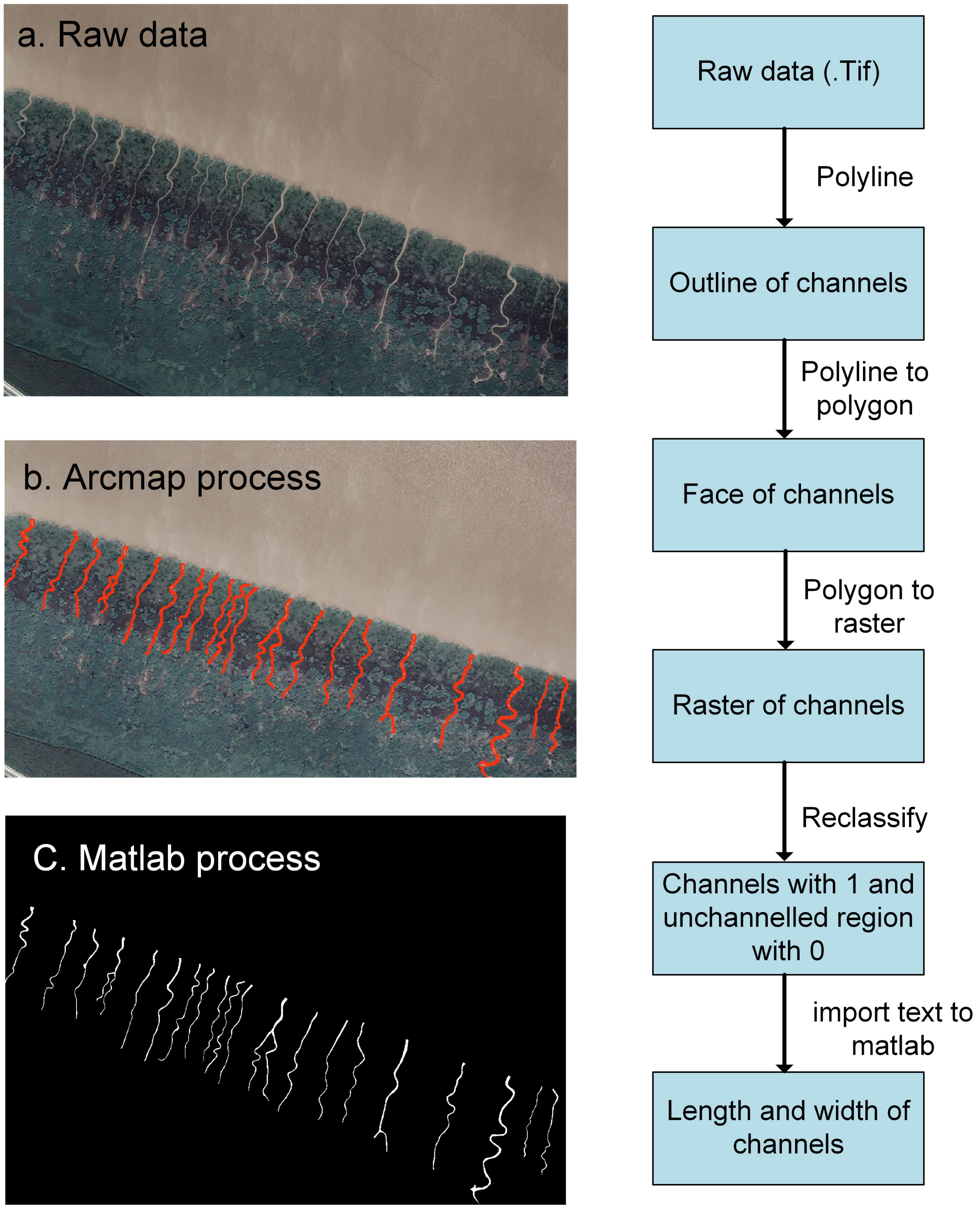

Figure. S4.1. Process of channel extraction. a Raw data from Google Earth, Image © 2024 CNES / Airbus. **b** Extracting channel pattern in Arcmap. **c** Analyse channel characteristics in Matlab.

First, The raw data of different areas is from Google Earth. Then, raw data was import in Arcmap. After polyline, polyline-to-polygon, polygon-to-raster, reclassification, the two classes is channels and unchannelled region. The resulting text was covered with value (channels with 1 and unchannelled region with 0). The length and width of channels were calculated in Matlab with the text.

Table. S4.1. Offshore tidal range, width of parent channel, and mean morphological parameters of parallel channels in selected locations.

	Tidal range (m)	Parent channel width (m)	Mean channel width (m)	Mean channel spacing (m)	Mean channel length (m)
Orne Estuary, France	7.1	324.3	4.3	58.6	232.6
Chongming Island, China	3	3355.0	5.3	117.4	513.0
Western Scheldt, Netherlands	4	1706.5	1.1	14.9	140.4
Hooge Plates, Netherlands	4.2	1565.5	1.1	20.2	63.5
Warbah Island, Kuwait	4	1460.5	5.5	176.6	609.0
Tillingham Marsh, UK	4.6	(connected to the open sea)	8.4	183.3	523.0
San Francisco Bay, USA	2.9	315.5	2.9	53.6	463.9
Ems-Dollard, Netherlands	2.9	270.1	3.3	109.0	174.8
Inhassunge Tidal Flats, Mozambique	3.7	245.0	4.1	112.1	260.3
Shoalwater Bay, Australia	6.9	266.1	10.8	329.9	1277.4
Meda River Delta, Australia	8.6	644.2	26.7	338.2	1115.7
Jiangsu Coast, China	5	(connected to the open sea)	238.45	3347.24	8081.11
Colorado River Delta, Mexico	6.9	29.5	8.5	98.5	499.4
Thengar Island, Bangladesh	3.9	3482.5	16.9	374.1	2840.4
Iranian Bay, Iran	4.2	153.6	7.8	132.2	388.6
Bahia Blanca, Argentina	2.5	26.7	3.0	16.4	125.9
Bahmanshir River Mouth, Iran	4	365.0	14.2	220.1	1646.5
Rio Cabelo da Valha, Brazil	4.5	666.3	23.5	606.1	1136.6
Taeryong River Delta, North Korea	7.5	1044.0	24.7	315.7	1280.3
Rio Cacine Channel, Guinea	4.5	191.8	15.2	439.3	890.6
Broome Tidal Flats, Australia	8.5	55.0	3.0	42.6	103.0
Scarped-shaped case, model	3.5	(connected to the open sea)	86.7	417.2	3437.2
Linear-shaped case, model	3.5	(connected to the open sea)	90.2	714.7	9983.2

Vegetated scarped-shaped case, model	3.5	(connected to the open sea)	45.1	344.6	3562.6

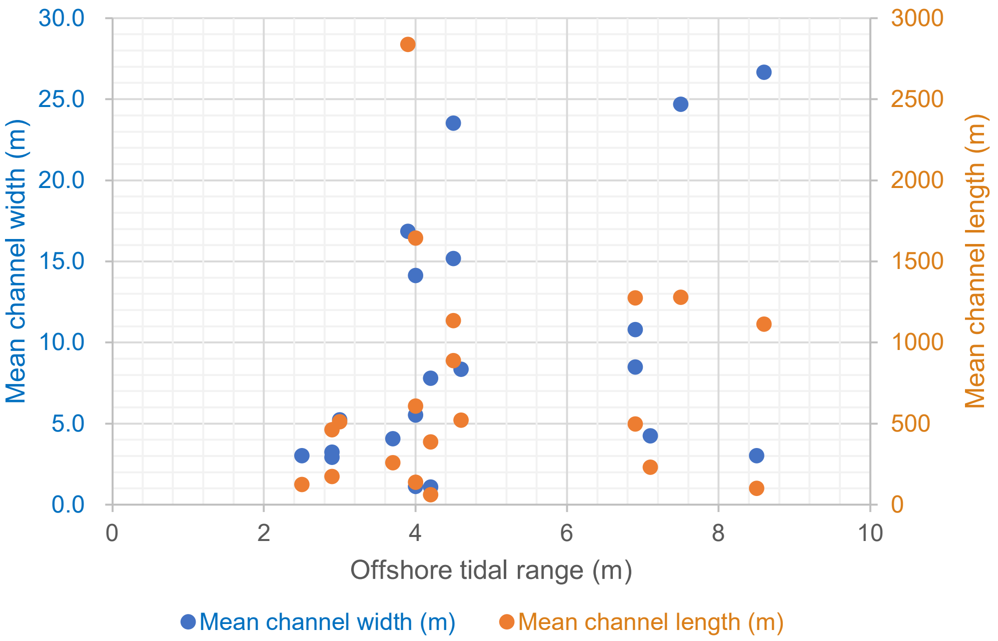

Figure. S4.2. The mean channel width and mean channel length of the 20 selected areas are plotted as a function of offshore tidal range.

Information about tidal range is acquired from ArcGIS map viewer: https://www.arcgis.com/apps/mapviewer/index.html?layers=d5354dea41b14f0689860bf4b2cf5e8a. Because of the lack of in-situ data, we can only find the offshore tidal range, which may be far away from the selected study areas. Therefore, the tidal range doesn't show a good relationship with the morphological parameters.

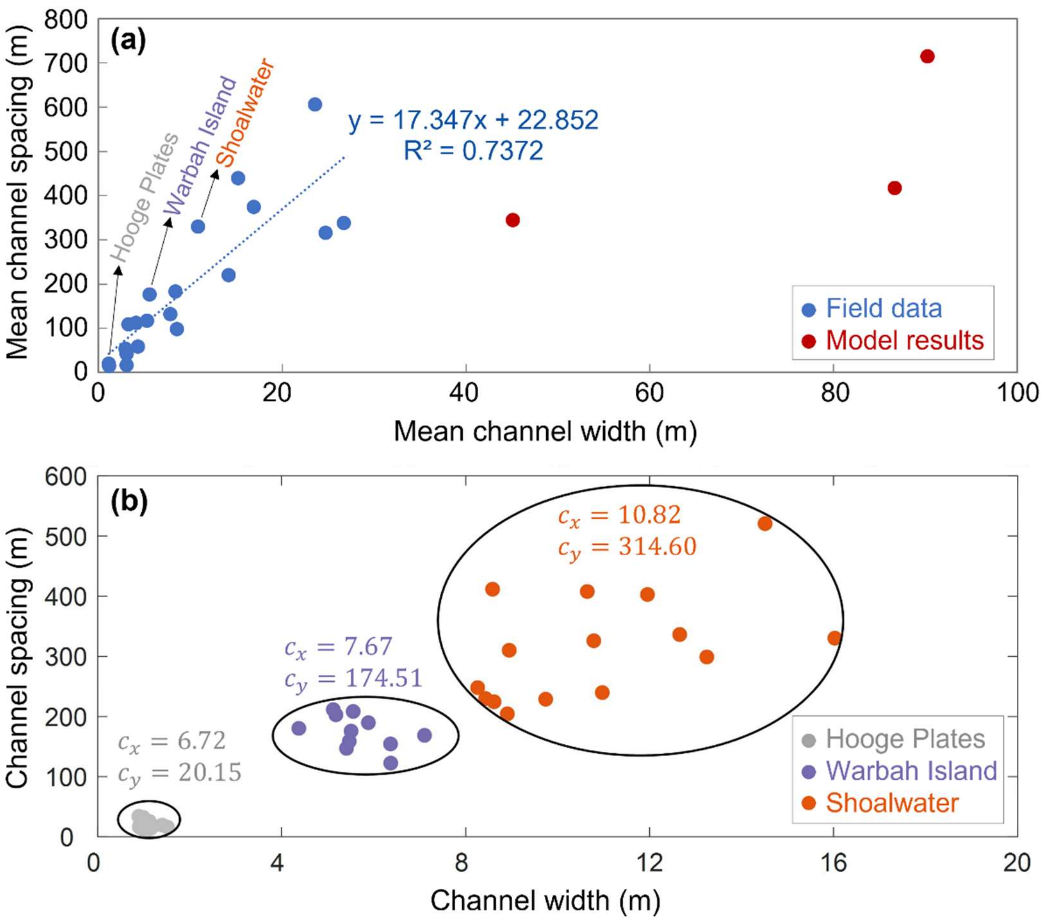

Figure. S4.3. The relationship between mean channel spacing and mean channel width of the parallel channel systems. a The mean channel spacings of the 20 selected areas and the model results are plotted as a function of mean channel width. **b** The channel spacings of 3 areas (Hooge Plates, Netherlands, Warbah Island, Kuwait, and Shoalwater Bay, Australia) are plotted as a function of channel width.

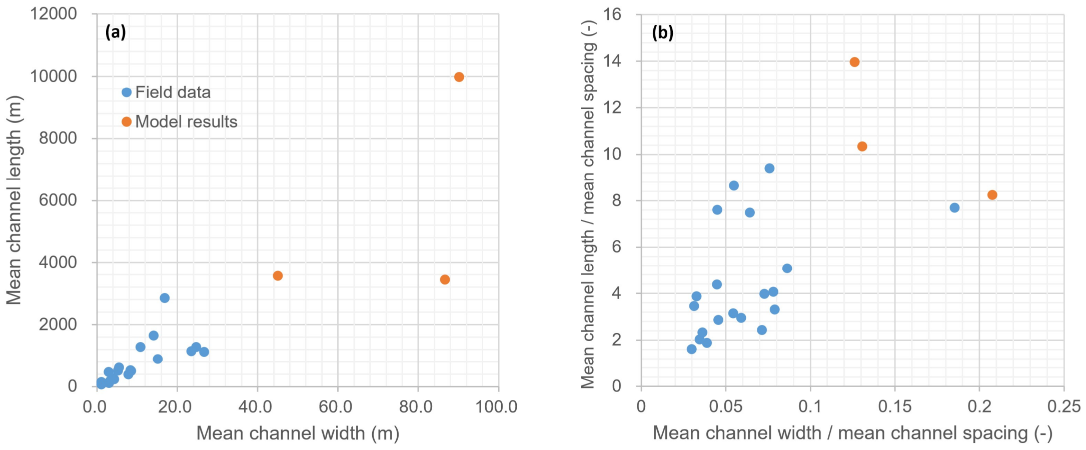

Figure. S4.4. The variations of channel length, width and spacing indifferent parallel channel systems. a The mean channel length of the 20 selected areas and model results are plotted as a function of mean channel width. **b** The relative channel length (calculated by the ratio of mean channel length and mean channel spacing) are

plotted as a function of relative channel width (calculated by the ratio of mean channel width and mean channel spacing).

Note that Fig. S4.2 - S4.4 do not include the situation of Jiangsu Coast, China, because the channel system in Jiangsu Coast is too large compared with other systems.
The mean channel spacing and mean channel width of the field channel systems approximately follow a linear relation (Fig. S4.3 a). However the model results do not agree with the trend of the tidal channels in the field, and the mean channel width is relatively larger. In Fig. S4.3 b, c_x and c_y are the dispersion coefficient of channel width and channel spacing, respectively. It shows that larger tidal channel systems also have greater degree of variation.
Due to the large grid in the model, the simulated tidal gully has a large scale, while the mean channel length and the mean channel width in both model and reality show a linear increasing trend (Fig. S4.4a). Fig. S4.4b shows the relative channel length as a function of relative channel width. Divided by the mean channel spacing, the two parameters express the degree of channelization per unit tidal flat area. Because of the distinct environmental conditions in different areas, the field data distributes dispersedly in Fig. S4.4b. Moreover, the model results show a larger trend than the field data, indicating that the simulated condition in the model tends to generate denser channel system.

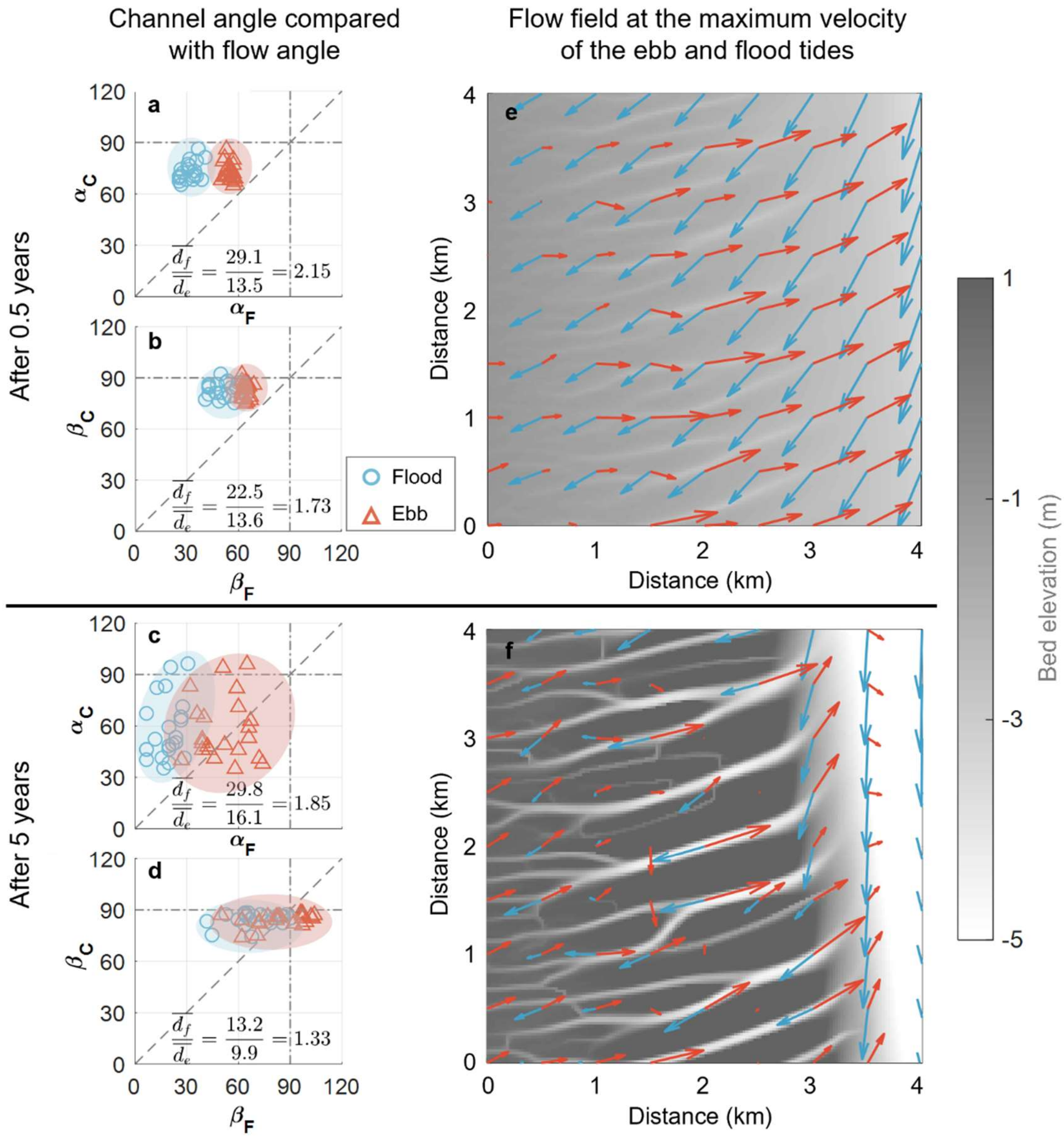

Figure. S4.5. Comparisons of channel angle and flow direction during flood and ebb tides. a-b Comparison between channel angle and flow direction during flood and ebb phases at the initial. **c-d** The channel angle and flow direction after 5 years. **e-f** The corresponding flow field at the maximum velocity of the ebb and flood tides.

The simulation was conducted on an initial bed surface where the upper platform has a slope of 0.08% and the transition point between the upper platform and the lower bank has an elevation of -3m. In Fig. S4.5 a-d, α_C and β_C are the connecting channel angles and overall channel angles, respectively. α_F and β_F are the flow angles measured at the roots and at the middle of the channel branches.

Fig. S4.5 shows that the flood flow angle is smaller than ebb flow angle, and the ebb flow is close to 90°. It indicates that the flood currents provide a stronger contribution on generating channel branches with small angles, while the direction of ebb currents is heavily influenced by gravity, resulting in a more perpendicular trend with the shorelines.

Section 5: Numerical solution of Poisson equation

The characteristic tidal flow field over tidal networks and flats is friction-dominated and can be approximated by a Poisson-type equation deduced from the shallow water equation. Therefore, the bending process of the streamline is subject to the balance between lateral water surface gradient and bed friction resistance, and can be explained by solving Poisson equation. Poisson equation can be expressed as (Di Silvio et al, 2010)[38]:

$$\frac{\partial^2 \eta}{\partial x^2} + \frac{\partial^2 \eta}{\partial y^2} = f(x) = \frac{\lambda a_0 2\pi}{h(x)^2 T} \tag{4}$$

where, $\eta(x, y)$ represents the fluctuation intensity of the free water surface relative to the mean water level. a_0 is the tidal amplitude, T is the tidal period, λ is a constant, and $h(x)$ is the average water depth (which does not vary with the (y) direction). With the boundary condition:

$$\eta|_{y=2000} = 0 \qquad \left.\frac{\partial \eta}{\partial y}\right|_{y=0} = 0 \qquad \left.\frac{\partial \eta}{\partial x}\right|_{x=0,x=800} = 0 \tag{5}$$

Here we simulated two cases with different bed surfaces (Fig. S5.1). In the two cases, the upper boundary is an open boundary, while the other three boundaries are closed boundaries. As the water flows into the basin from the upper boundary, there are abrupt changes in the flow direction in the case with sudden changes in bed slope. In cases where the bed slope is uniform, the changes of flow direction are also gradual.

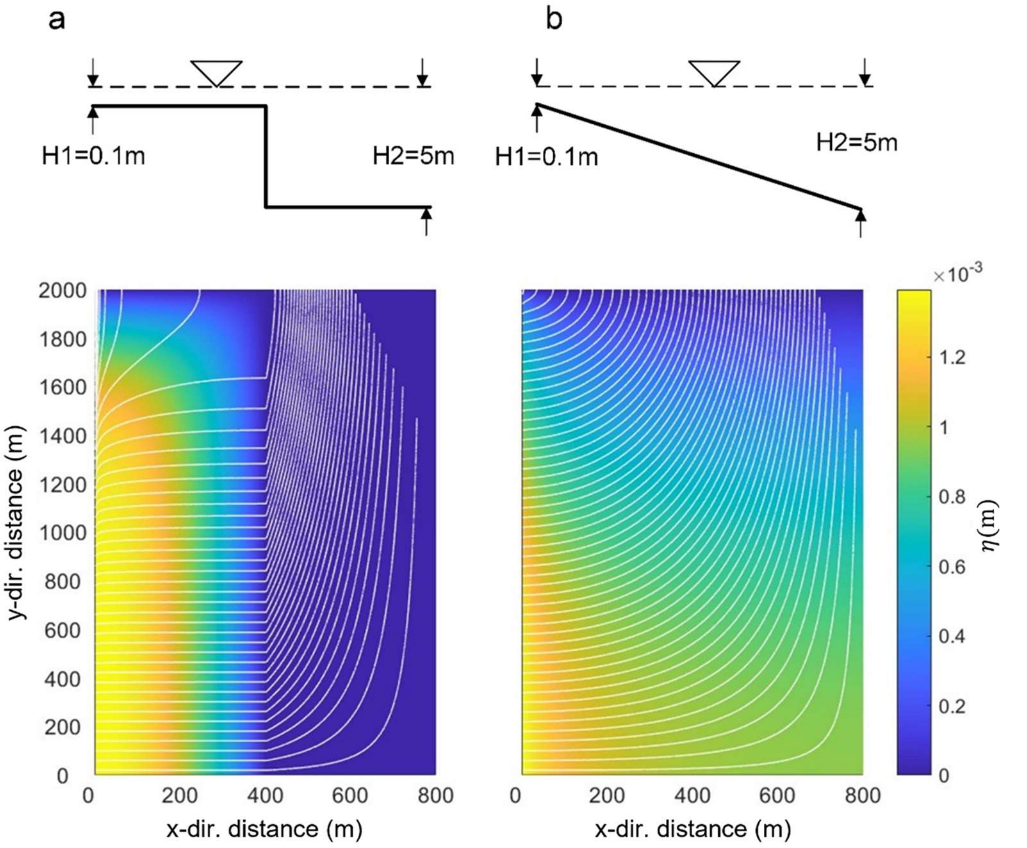

Figure. S5.1. Comparison of flow lines between cases with abruptly-changing slope and uniform slope. a The case with abruptly-changing slope. **b** The case with uniform slope.

Supplementary References

1. Ou, Y. & Liu, C. Analysis on sediment transport patterns and sediment sources of north branch of Changjiang estuary. *J Hydraul Eng*, 79-84 (2002).
2. Liu, B., Zhang, G., Li, Y. & Li, Z. Sediment Characteristics and Transport Trend in North Branch and Offshore Area of Yangtze Estuary in the Last 30 Years. *Resources and Environment in the Yangtze Basin*. **27**, 2328-2338 (2018).
3. Baby, S. Information research on coastal morphological environment of Kuwait, organizations, role and coastal legislations. *Emirates Journal for Engineering Research*. **2**, 7-24 (2011).
4. Al-Ghadban, A. N. Holocene sediments in a shallow bay, southern coast of Kuwait, Arabian Gulf. *Mar. Geol.* **92**, 237-254 (1990).
5. Bolle, A., Bing Wang, Z., Amos, C. & De Ronde, J. The influence of changes in tidal asymmetry on residual sediment transport in the Western Scheldt. *Cont. Shelf Res.* **30**, 871-882 (2010).
6. Stark, J., Smolders, S., Meire, P. & Temmerman, S. Impact of intertidal area characteristics on estuarine tidal hydrodynamics: A modelling study for the Scheldt Estuary. *Estuarine, Coastal and Shelf Science*. **198**, 138-155 (2017).
7. Ridderinkhof, H., van der Ham, R. & van der Lee, W. Temporal variations in concentration and transport of suspended sediments in a channel‐flat system in the Ems-Dollard estuary. *Cont. Shelf Res.* **20**, 1479-1493 (2000).
8. Barnard, P. L. et al. Integration of bed characteristics, geochemical tracers, current measurements, and numerical modeling for assessing the provenance of beach sand in the San Francisco Bay Coastal System. *Mar. Geol.* **336**, 120-145 (2013).
9. Zigic, S., Makarynskyy, O., Langtry, S. & Westbrook, G. A Numerical Modelling Study for the Proposed Increase in Barramundi Production, Cone Bay, Western Australia. *Proceedings of the 11Th Estuarine and Coastal Modelling Conference*. **41121**, 388 (2010).
10. Lazarus, E. D. & Constantine, J. A. Generic theory for channel sinuosity. *Proceedings of the National Academy of Sciences*. **110**, 8447-8452 (2013).
11. Finotello, A. et al. Field migration rates of tidal meanders recapitulate fluvial morphodynamics. *Proceedings of the National Academy of Sciences*. **115**, 1463-1468 (2018).
12. Rinaldo, A., Fagherazzi, S., Lanzoni, S., Marani, M. & Dietrich, W. E. Tidal networks: 3. Landscape‐forming discharges and studies in empirical geomorphic relationships. *Water Resour. Res.* **35**, 3919-3929 (1999).
13. Zhao, F., Li, Z., Li, J. & Chen, W. Mechanism of water and suspended sediment transport from neap tide to spring tide in North Branch of Changjiang Estuary. *Journal of Sediment Research*, 55-62 (2013).
14. Gong, Z. et al. Mechanisms underlying the dynamic evolution of an open-coast tidal flat-creek system: Ⅱ: influence of tidal range. *Advances in Water Science*. **2**, 231-239 (2017).
15. Yang, H., Su, T., Kong, D. & Dai, X. Grain-size characteristics of the inter-tidal flat sediments of Shanghai nonresident islands. *Journal of Shanghai Ocean University*. **21**, 257-264 (2012).
16. Li, Z., Gao, S. & Shen, H. Suspended sediment concentration profiles and grain size distribution patterns over the Dafeng tidalflat. *Journal of Sediment Research*, 62-70 (2006).
17. Khalaf, F. I., Al-Ghadban, A., Al-Saleh, S. & Al-Omran, L. Sedimentology and mineralogy of Kuwait Bay bottom sediments, Kuwait-Arabian Gulf. *Mar. Geol.* **46**, 71-99 (1982).
18. Khalaf, F., Al-Bakri, D. & Al-Ghadban, A. Sedimentological characteristics of the surficial sediments of the Kuwaiti marine environment, northern Arabian Gulf. *Sedimentology*. **31**, 531-545 (1984).
19. Reed, D. J. Sediment dynamics and deposition in a retreating coastal salt marsh. *Estuarine, Coastal and Shelf Science*. **26**, 67-79 (1988).
20. Reed, D. J., Stoddart, D. R. & Bayliss-Smith, T. P. Tidal Flows and Sediment Budgets for a Salt-Marsh System, Essex, England. *Vegetatio*. **1-3**, 375-380 (1985).
21. Brooks, H., Moller, I., Spencer, T., Royse, K. & Price, S. J. Geotechnical Properties of Salt Marsh and Tidal Flat Substrates at Tillingham, Essex, UK. *Coastal Engineering Proceedings*. **1**, 55 (2018).
22. Callaghan, D. P. et al. Hydrodynamic forcing on salt-marsh development: Distinguishing the relative importance of waves and tidal flows. *Estuarine, Coastal and Shelf Science*. **89**, 73-88 (2010).
23. Van Den Berg, J. H., Jeuken, C. J. & Van der Spek, A. J. Hydraulic processes affecting the morphology and evolution of the Westerschelde estuary. *Estuarine Shores: Evolution, Environments and Human Alterations*, 157-184 (1996).
24. Willemsen, P. W. J. M. et al. Quantifying Bed Level Change at the Transition of Tidal Flat and Salt Marsh: Can We Understand the Lateral Location of the Marsh Edge? *Journal of Geophysical*

Research: Earth Surface. **123**, 2509-2524 (2018).

25. Folmer, E. O. et al. Space－time analyses of sediment composition reveals synchronized dynamics at all intertidal flats in the Dutch Wadden Sea. *Estuarine, Coastal and Shelf Science*. **285**, 108308 (2023).
26. Ruhl, C. A., Schoellhamer, D. H., Stumpf, R. P. & Lindsay, C. L. Combined Use of Remote Sensing and Continuous Monitoring to Analyse the Variability of Suspended-Sediment Concentrations in San Francisco Bay, California. *Estuarine, Coastal and Shelf Science*. **53**, 801-812 (2001).
27. Kranck, K. & Milligan, T. G. Characteristics of suspended particles at an 11-hour anchor station in San Francisco Bay, California. *Journal of Geophysical Research*. **97**, 11373-11382 (1992).
28. Semeniuk, V., Brocx, M., Western, A. M. S. I., Brocx, M. & Meney, K. King Sound and the tide-dominated delta of the Fitzroy River; their geoheritage values. *Journal of the Royal Society of Western Australia*. **94**, 151-160 (2011).
29. Blanchet, H. et al. Multiscale patterns in the diversity and organization of benthic intertidal fauna among French Atlantic estuaries. *J. Sea Res.* **90**, 95-110 (2014).
30. Keulen, M. V. & Borowitzka, M. A. Seasonal variability in sediment distribution along an exposure gradient in a seagrass meadow in Shoalwater Bay, Western Australia. *Estuarine, Coastal and Shelf Science*. **57**, 587-592 (2003).
31. Zhou, Z. et al. Morphodynamics of river－influenced back－barrier tidal basins: the role of landscape and hydrodynamic settings. *Water Resour. Res.* **50**, 9514-9535 (2014).
32. Zhou, Z., Ye, Q. & Coco, G. A one-dimensional biomorphodynamic model of tidal flats: Sediment sorting, marsh distribution, and carbon accumulation under sea level rise. *Adv. Water Resour.* **93**, 288-302 (2016).
33. Soulsby, R. Dynamics of marine sands.; 1997.
34. van der Wegen, M. & Roelvink, J. A. Long－term morphodynamic evolution of a tidal embayment using a two－dimensional, process－based model. *Journal of Geophysical Research: Oceans*. **113**, (2008).
35. Zhang, Q. et al. The Role of Surges During Periods of Very Shallow Water on Sediment Transport Over Tidal Flats. *Front. Mar. Sci.* **8**, (2021).
36. Zhang, Q. et al. Velocity and sediment surge: What do we see at times of very shallow water on intertidal mudflats? *Cont. Shelf Res.* **113**, 10-20 (2016).
37. Dai, Z., Fagherazzi, S., Mei, X., Chen, J. & Meng, Y. Linking the infilling of the North Branch in the Changjiang (Yangtze) estuary to anthropogenic activities from 1958 to 2013. *Mar. Geol.* **379**, 1-12 (2016).
38. Di Silvio, G., Dall Angelo, C., Bonaldo, D. & Fasolato, G. Long-term model of planimetric and bathymetric evolution of a tidal lagoon. *Cont. Shelf Res.* **30**, 894-903 (2010).
