# Supplementary material for: Cross-shore parallel tidal channel systems formed by alongshore currents
Source: Nat Commun. 2024 Jun 3;15:4732. doi: 10.1038/s41467-024-49176-2 (PMC11148050; doi:10.1038/s41467-024-49176-2)
Supplement: Supplementary file 3 — Description of Additional Supplementary Files [file 41467_2024_49176_MOESM3_ESM.pdf]

Description of Additional Supplementary Files

File name: Supplementary Data 1

Description: Morphological characteristics of parallel tidal channel systems around the world**.** The data regarding the worldwide parallel channels, including channel angles, length, width, and spacing.
