# Supplementary material for: Cross-shore parallel tidal channel systems formed by alongshore currents
Source: Nat Commun. 2024 Jun 3;15:4732. doi: 10.1038/s41467-024-49176-2 (PMC11148050; doi:10.1038/s41467-024-49176-2)
Supplement: Supplementary file 5 — Reporting Summary [file 41467_2024_49176_MOESM5_ESM.pdf]

nature portfolio

Corresponding author(s): Liang Geng; Fan Xu

Last updated by author(s): May 12, 2024

Reporting Summary

Nature Portfolio wishes to improve the reproducibility of the work that we publish. This form provides structure for consistency and transparency in reporting. For further information on Nature Portfolio policies, see our Editorial Policies and the Editorial Policy Checklist.

Statistics

For all statistical analyses, confirm that the following items are present in the figure legend, table legend, main text, or Methods section.

n/a	Confirmed	
[x]	[ ]	The exact sample size (n) for each experimental group/condition, given as a discrete number and unit of measurement
[x]	[ ]	A statement on whether measurements were taken from distinct samples or whether the same sample was measured repeatedly
[x]	[ ]	The statistical test(s) used AND whether they are one- or two-sided<br>*Only common tests should be described solely by name; describe more complex techniques in the Methods section.*
[x]	[ ]	A description of all covariates tested
[x]	[ ]	A description of any assumptions or corrections, such as tests of normality and adjustment for multiple comparisons
[ ]	[x]	A full description of the statistical parameters including central tendency (e.g. means) or other basic estimates (e.g. regression coefficient) AND variation (e.g. standard deviation) or associated estimates of uncertainty (e.g. confidence intervals)
[x]	[ ]	For null hypothesis testing, the test statistic (e.g. F, t, r) with confidence intervals, effect sizes, degrees of freedom and P value noted<br>*Give P values as exact values whenever suitable.*
[x]	[ ]	For Bayesian analysis, information on the choice of priors and Markov chain Monte Carlo settings
[x]	[ ]	For hierarchical and complex designs, identification of the appropriate level for tests and full reporting of outcomes
[x]	[ ]	Estimates of effect sizes (e.g. Cohen's d, Pearson's r), indicating how they were calculated

Our web collection on statistics for biologists contains articles on many of the points above.

Software and code

Policy information about availability of computer code

Data collection	The model Delft3D is an open-source code available online (at https://oss.deltares.nl). The model set-up is available at the repository Zenodo https://zenodo.org/records/10814687)
Data analysis	MATLAB 2022a

For manuscripts utilizing custom algorithms or software that are central to the research but not yet described in published literature, software must be made available to editors and reviewers. We strongly encourage code deposition in a community repository (e.g. GitHub). See the Nature Portfolio guidelines for submitting code & software for further information.

Data

Policy information about availability of data

All manuscripts must include a data availability statement. This statement should provide the following information, where applicable:

- Accession codes, unique identifiers, or web links for publicly available datasets
- A description of any restrictions on data availability
- For clinical datasets or third party data, please ensure that the statement adheres to our policy

The data regarding the worldwide parallel channels are available as supplementary material (Supplementary Data.xlsx).

Research involving human participants, their data, or biological material

Policy information about studies with human participants or human data. See also policy information about sex, gender (identity/presentation), and sexual orientation and race, ethnicity and racism.

Reporting on sex and gender	This information has not been collected.
Reporting on race, ethnicity, or other socially relevant groupings	No socially constructed or socially relevant categorization variable are used.
Population characteristics	See above
Recruitment	Our research does not involve human participants, their data, or biological material.
Ethics oversight	Our research does not involve human participants, their data, or biological material.

Note that full information on the approval of the study protocol must also be provided in the manuscript.

Field-specific reporting

Please select the one below that is the best fit for your research. If you are not sure, read the appropriate sections before making your selection.

☐ Life sciences ☐ Behavioural & social sciences ☒ Ecological, evolutionary & environmental sciences

For a reference copy of the document with all sections, see nature.com/documents/nr-reporting-summary-flat.pdf

Ecological, evolutionary & environmental sciences study design

All studies must disclose on these points even when the disclosure is negative.

Study description	The study conducts a comprehensive investigation into the formation and evolution of parallel tidal channel systems, influenced primarily by alongshore tidal currents and bed topography across various global coastal settings. It utilizes a mixed-method approach, combining remote sensing imagery analysis and morphodynamic simulations via Delft3D software. The research quantitatively examines 275 parallel channels from 21 diverse sites, focusing on channel orientation and morphology. Key variables include channel angles, bed elevation changes, and tidal range, with statistical analyses employing measures such as means, medians, and distribution patterns to elucidate the environmental interactions shaping these tidal channel systems.
Research sample	In our study, the samples consist of parallel tidal channels as observed in satellite images.
Sampling strategy	In our approach to studying tidal channels, we did not employ a specialized sampling strategy; instead, our methodology focused on collecting as much data as possible from around the world. Although we were not able to collect a complete global dataset, our study still boasts the largest dataset compared to existing research in this field.
Data collection	We first collect available satellite images of tidal channels, then we utilized MATLAB to delineate and quantify the characteristics of the tidal channels.
Timing and spatial scale	The timing of our sampling is contingent upon the capture dates of the imagery used. It's important to emphasize that the channel systems we observed have been consistently present for over five years, as can be clearly discerned from remote sensing images spanning multiple years. The morphological features of the channels that we focus on are also relatively stable. Therefore, the specific timing of data collection is not crucial to the results of our study.<br>The data encompass a wide geographical scope, with parallel channels analyzed across 21 different sites worldwide, including diverse geomorphological settings such as estuaries, lagoons, and open coasts. This broad spatial scale ensures that the findings are applicable to a variety of coastal environments, enhancing the generalizability of the study results.
Data exclusions	No data were excluded.
Reproducibility	All simulations are reproducible.
Randomization	We sampled the data randomly.
Blinding	In our study of naturally self-organizing phenomena, blinding was not employed as it is generally not applicable or necessary for this type of research.

Did the study involve field work? ☐ Yes ☒ No

Reporting for specific materials, systems and methods

We require information from authors about some types of materials, experimental systems and methods used in many studies. Here, indicate whether each material, system or method listed is relevant to your study. If you are not sure if a list item applies to your research, read the appropriate section before selecting a response.

Materials & experimental systems

n/a	Involved in the study
☒	☐ Antibodies
☒	☐ Eukaryotic cell lines
☒	☐ Palaeontology and archaeology
☒	☐ Animals and other organisms
☒	☐ Clinical data
☒	☐ Dual use research of concern
☒	☐ Plants

Methods

n/a	Involved in the study
☒	☐ ChIP-seq
☒	☐ Flow cytometry
☒	☐ MRI-based neuroimaging

Plants

Seed stocks	n/a
Novel plant genotypes	n/a
Authentication	n/a
